# Patient preferences in the benefit–risk assessment of atopic dermatitis treatments in China: A nationwide survey

**DOI:** 10.1016/j.jdin.2025.11.011

**Published:** 2025-11-27

**Authors:** Jingyi Yang, Yang Liu, Hengjing Li, Yumei Ge, Chen Yin, Yun Zhai, Cong Zhao, Siyuan Qian, Xiaoyuan Chen, Songmei Xie

**Affiliations:** a Center for Drug Evaluation, National Medical Products Administration, Beijing, China; b Tsinghua Clinical Research Institute, School of Basic Medical Sciences, Tsinghua University, Beijing, China; c Clinical Trial Center for Drugs and Medical Devices, Beijing Tsinghua Changgung Hospital, School of Clinical Medicine, Tsinghua University, Beijing, China

**Keywords:** atopic dermatitis, benefit-risk assessment, patient experience data, patient-focused drug development, regulatory sciences

## Abstract

**Background:**

Understanding patient preferences is critical for patient-focused drug development, especially in chronic conditions like atopic dermatitis, where treatment benefit–risk profiles vary widely.

**Objective:**

To evaluate treatment priorities, risk tolerance, and benefit–risk acceptance among Chinese patients with atopic dermatitis.

**Methods:**

A nationwide, cross-sectional online survey was conducted between February and July 2023. Patients with physician-diagnosed atopic dermatitis or their caregivers were recruited via major dermatology hospitals and patient communities. The survey evaluated treatment goals, unacceptable adverse events, and willingness to accept risks under scenarios of near-complete versus partial recovery.

**Results:**

Of 2120 respondents, 1948 valid responses were analyzed. Top treatment goals were reducing flare-ups (84%), preventing complications (61%), and relieving itch and pain (52%). Most unacceptable adverse events were serious infections (67%) and malignancies (65%). Patients showed increased risk tolerance when treatments promised near-complete recovery (15% fully accepted <0.1% risk of severe reactions) versus partial improvement (10%). Risk tolerance was lower among patients under 18 years and those with mild-to-moderate disease.

**Limitations:**

Self-reported data and online recruitment may limit generalizability.

**Conclusion:**

Chinese patients are more willing to accept treatment risks when greater benefits are expected. Preferences vary by age and severity, supporting more personalized treatment discussions and decisions.

**Capsule Summary**

• This nationwide survey is the first to assess treatment preferences and risk tolerance among patients with eczema in China, revealing age- and severity-based differences in decision-making.
• Dermatologists/policy makers should consider individual patient values and risk tolerance when discussing treatment options and selecting therapies, especially in younger or milder cases.

## Introduction

Atopic dermatitis (AD) is a chronic, relapsing skin disease characterized by intense itching and recurrent rashes,^1^ with significant physical and psychological burden.^2^^,^^3^ Prevalence reaches 15% to 20% in children globally^1^ and 30.5% among Chinese infants under 12 months.^4^ AD commonly begins in childhood but may persist or start in adults. Treatments, primarily anti-inflammatory agents or immunosuppressants,^5^ are often off-label and suboptimal, with patterns varying geographically.

AD’s burden differs by age, severity, region, and treatment.^6^, ^7^, ^8^, ^9^ Greater severity is associated with worse clinical, quality of life, psychosocial, and economic outcomes.^10^ China reports the largest adult and pediatric patient populations globally.^11^

In China, approved systemic treatments include the IL-4/IL-13 inhibitor dupilumab and oral JAK inhibitors (abrocitinib, upadacitinib). Topical options include corticosteroids, calcineurin inhibitors, and the PDE-4 inhibitor crisaborole. Recently, benvitimod (aryl hydrocarbon receptor agonist) and stapokibart (IL-4/IL-13 inhibitor) were also approved. However, some mechanisms of action raise safety concerns, for example, the risks of serious infections or malignancy.^12^

Clinical research in China is expanding,^13^ with 339 China-registered multi-regional clinical trials launched across all therapeutic areas as of 2024.^14^ Dermatological trials significantly increased, especially innovative drugs,^15^^,^^16^ presenting opportunities to align drug development with patient needs.

Understanding patients’ benefit-risk preferences is essential for drug development and regulation. While such studies exist internationally^17^^,^^18^ and are integrated into regulatory review,^19^^,^^20^ none are from China. Cultural, ethnic, and health care system differences may affect applicability. This is the first study to assess Chinese AD patients’ benefit-risk acceptance for clinical and regulatory considerations.

## Methods

From 3 February to 7 July 2023, participants were recruited via electronic invitations from major hospitals (see Supplementary Material, available via Mendeley at https://data.mendeley.com/datasets/xzddbfkj3c/1) and through “China AD Home” and hospital-based patient groups across China. Eligible respondents were patients with physician-confirmed AD or their caregivers.

The anonymous survey was conducted via Wenjuanxing, a secure online platform. No identifiers were collected. Survey purpose, procedures, and voluntary participation was explained upfront; informed consent implied upon completion. Reasons for nonparticipation were not collected.

As this study involved no identifiable personal data, interventions, or specimens, ethics review was not required.

### Survey design

This cross-sectional online survey assessed treatment preferences and experiences of AD patients in China. The survey instrument was developed through literature review and expert input from regulators, dermatologists, and researchers. It included 4 sections: demographics, clinical characteristics, psychosocial impact, and treatment preferences, using multiple-choice questions and 5-/3-point Likert scales.

### Study outcome

The primary outcomes were the proportions of participants willing to accept treatment risks targeting (1) near-complete recovery and (2) partial improvement of AD across varying risk profiles. Secondary outcomes included acceptance for specific adverse events and benefits.

### Statistical analysis

Respondents self-reporting a physician-confirmed AD diagnosis at a medical institution were valid and included in the final analysis.

We ranked frequencies of 16 adverse events and 7 treatment benefits using multiple-response analysis, comparing distributions by age group (<18 vs ≥18 years). To describe the benefit-risk acceptance, we calculated acceptance levels (fully, partially, or not accepted) across 4 risk scenarios under near-complete and partial recovery. Results were visualized by age and disease severity (mild-to-moderate vs severe).

Pearson Chi-square tests were performed to ascertain statistical significance of between group differences. Two-sided statistical tests were employed with a significance level set at 0.05 in all comparisons. All statistical analyses were executed using SAS version 9.4 (SAS Institute Inc).

## Results

### Cohort characteristics

Of 2120 respondents, 1948 valid responses were included (92%). Among 172 excluded responses, 116 (5%) reported no physician-confirmed diagnosis of AD, and 56 (3%) reported not knowing or not remembering such a diagnosis.

Among the 1948 responses, participants were drawn from all major regions (Supplementary Fig 1, available via Mendeley at https://data.mendeley.com/datasets/xzddbfkj3c/1), with East China contributing the most (630, 32%). 958 participants were female. 607 (31%) were below 18 years, including 402 children under 11. 761 (39.1%) responses were caregivers-completed; 596 (30.6%) for patients under 18 years. By age group, caregiver-completed responses proportions were: <11 years, 94.78%; 11-17 years, 84.88%; ≥18 years, 15.36%. Disease severity was reported as mild (37%), moderate (46%), and severe (17%). Upper and lower limbs were the most affected areas (both 64%).

AD had moderate or greater impact on daily life in 1125 participants (58%), with 69% experienced moderate-to-severe sleep disruption. Most bothersome symptoms were itching (68%), skin dryness/tightness (34%), sleep disturbances (28%), thickened plaques (24%), and oozing or crusting (23%). Demographic features and comorbidities were similar to previous reports.^8^^,^^21^

Topical prescription treatments were used by 84% of participants, systemic therapies by 66%, and adjunctive approaches by 60%. The most common topical agents were corticosteroids (68%), calcineurin inhibitors (52%), and crisaborole (25%). Dupilumab was the most frequently ever used systemic therapy (62%), reported by 64% of mild, 58% of moderate, and 71% of severe patients. Moisturizers (53%), traditional Chinese medicine and dietary changes (57%), and lifestyle modifications (35%) were the most reported adjunctive therapies. Overall, 55% patients used both topical and systemic therapies, while only 4.2% had used neither. Additionally, 59% had used at least 2 topical and 34% had 2 or more systemic therapies, indicating widespread use of combination or sequential strategy.

### Preference on current treatment modalities

47% patients perceived their disease as well controlled, 11% as very well controlled, while 38% reported poor or no control. Perceived control declined with increasing severity: 17% of mild, 49% of moderate, and 55% of severe AD reported poor or no control. When asked the most effective treatments (multiple responses allowed), patients most frequently cited basic skincare (60%), followed by topical medications (44%, including crisaborole, tacrolimus, and corticosteroids), injectable biologics (40%), and lifestyle modifications (22%, such as changes in environmental exposures, sleep patterns, and diet). A higher proportion of patients with severe disease considered injectable biologics effective compared to topical therapies, and this difference was less pronounced among patients with mild-to-moderate disease.

### Perceived barriers to treatment

The most reported barriers were concerns about long-term medication use (46%), financial burden (36%), time-consuming treatment (32%), inconvenience (27%), and adverse effects (20%). These barriers were more common among patients with moderate-to-severe disease, particularly regarding poor efficacy, long-term use, and cost.

Among 675 patients who discontinued or changed treatment, main reasons were lack of efficacy (45%), long-term use concerns (37%), and perceived resolution of symptoms (30%). Of 1323 corticosteroids users, 34% experienced severe or prolonged flares after discontinuation.

### Preferences for risks and clinical benefits of AD treatments

As shown in Table I, severe infections (eg, pneumonia, cellulitis) were most concerning (67%), followed by malignancies, lymphoproliferative diseases, and gastrointestinal perforation (65%). Cardiovascular events (58%), skin reactions (56%), thrombosis (53%), and allergic reactions (52%) were also viewed as unacceptable. A similar trend was observed across age groups, though skin reactions and allergic reactions ranked higher among younger participants.

**Table I:** Distribution of unacceptable adverse events across age groups

Grouped items (unacceptable adverse reactions)	Age ≥18y *N* (%)	Age <18y *N* (%)	Total	*P* value∗
Serious infections, such as infectious pneumonia, cellulitis, etc.	891 (68)	414 (32)	1305	.03
Malignant tumors and lymphoproliferative disorders, gastrointestinal perforation	873 (69)	389 (31)	1262	
Allergic reactions	671 (66)	350 (34)	1021	
Thrombosis	692 (68)	332 (32)	1024	
Cardiovascular adverse events, such as nonfatal myocardial infarction	779 (69)	350 (31)	1129	
Skin reactions, such as irritation, pigmentation, skin atrophy, etc.	719 (66)	366 (34)	1085	
Osteoporosis	567 (66)	292 (34)	859	
Neutropenia	446 (64)	256 (36)	702	
Lymphopenia	451 (64)	259 (36)	710	
Anemia	415 (63)	247 (37)	662	
Dyslipidemia	455 (64)	252 (36)	707	
Elevated liver enzymes	489 (65)	260 (35)	749	
Hyperglycemia	464 (65)	248 (35)	712	
Hypertension	472 (66)	241 (34)	713	
Weight gain	473 (69)	211 (31)	684	
Others	34 (58)	25 (42)	59	

∗ Pearson’s Chi-square test.

**Fig 1:**
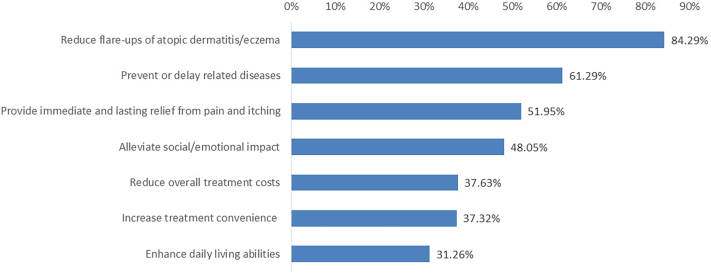
Primary treatment benefits considered by patients. Patients selected top benefits when choosing treatments for atopic dermatitis without a complete cure. Flare-up reduction (84%) and disease prevention (61%) ranked highest.

Most participants considered reducing flare-ups of AD as the most important benefits when selecting treatments not offering a complete cure (84%), followed by preventing/delaying related diseases (61%), and providing immediate and lasting relief from pain and itching (52%) (Fig 1).

### Benefit-risk acceptance

Table II shows that patients’ risk tolerance varied by treatment outcome and event severity. For near-complete recovery, 15% fully accepted <0.1% risk of severe adverse events, dropping to 10% when the risk increased to 0.1% to 1%. Acceptance was lowest (9%) for risks affecting quality of life (1% to 10%) and highest (22%) for minor adverse events (>10%). Overall acceptance was lower in partial recovery scenarios, with only 10% fully accepting <0.1% risk and 7% accepting 0.1% to 1% risk. These findings highlight patients' greater willingness to accept higher risks for minor adverse reactions, especially when aiming for near-complete recovery.

**Table II:** Risk acceptance for treatments targeting near-complete or partial recovery of atopic dermatitis/eczema

	Fully accept *N* (%)	Partially accept *N* (%)	Do not accept *N* (%)	*P* value∗
Targeting near-complete recovery				
Probability <0.1%, severe adverse reactions, eg, malignant tumors	296 (15)	557 (29)	1095 (56)	<.01
Probability 0.1% to 1%, severe adverse reactions, eg, malignant tumors	195 (10)	429 (22)	1324 (68)	
Probability 1% to 10%, adverse reactions affecting quality of life, eg, lung infections	176 (9)	613 (31)	1159 (60)	
Probability >10%, minor impact adverse reactions, eg, elevated transaminase levels	427 (23)	891 (46)	630 (32)	
Targeting partial recovery				
Probability <0.1%, severe adverse reactions, eg, malignant tumors	185 (10)	464 (24)	1299 (67)	<.01
Probability 0.1% to 1%, severe adverse reactions, eg, malignant tumors	144 (7)	348 (18)	1456 (75)	
Probability 1% to 10%, adverse reactions affecting quality of life, eg, lung infections	134 (7)	519 (27)	1295 (66)	
Probability >10%, minor impact adverse reactions, eg, elevated transaminase levels	269 (14)	833 (43)	846 (43)	

∗ Pearson’s Chi-square test.

**Fig 2:**
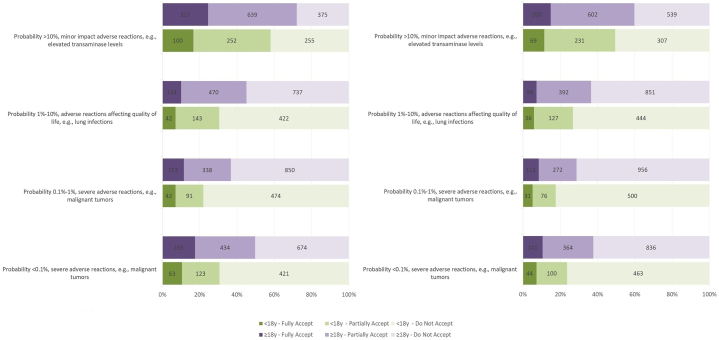
Risk acceptance by age group and expected treatment outcome. Risk acceptance for treatments targeting near-complete recovery (left) versus partial improvement (right) of atopic dermatitis, stratified by age group (<18 years vs ≥18 years). Patients below 18 years (or their caregivers) were generally more risk-averse across all scenarios, especially for treatments associated with even minor adverse events.

**Fig 3:**
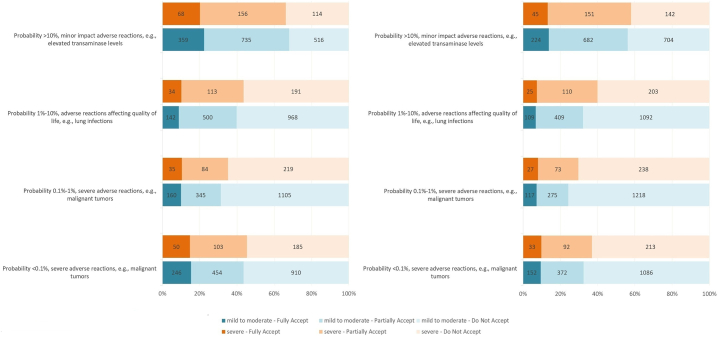
Risk acceptance by disease severity and expected treatment outcome. Risk acceptance for treatments targeting near-complete recovery (left) versus partial improvement (right) of atopic dermatitis (AD), stratified by disease severity (mild to moderate vs severe). Patients with severe AD showed greater willingness to accept risks, particularly for treatments promising near-complete recovery.

Patients with mild-to-moderate disease and those under 18 were more risk-averse (Figs 2 and 3). Patients with severe disease were more willing to accept both moderate but frequent and severe but rare adverse reactions than those with mild-to-moderate disease. Acceptance of minor, frequent adverse events was similar across severity groups. Risk tolerance differences by severity were less pronounced under partial recovery scenarios. When comparing across age groups, younger patients (or their representatives) were less risk-tolerant, even for minor impact reactions. This age-related difference was less evident for treatment targeting partial recovery.

## Discussions

To our knowledge, this is the first nationwide survey to evaluate treatment preferences and benefit-risk acceptance among the Chinese AD population. Specifically, we assessed patients’ priorities and willingness to accept different levels of treatment risk under varying expected benefits.

### Unmet therapeutic needs of patients

Patient preference data clarify which outcomes matter most and where current treatments fall short. Current treatments primarily target lesion improvement, yet no cure exists, and long-term medication is often required. Many patients or caregivers reported poor control and treatment discontinuation. In our survey, 36% reported poor control, and 35% had changed or stopped treatment. Reducing flare frequency was the most desired outcome.

These findings echo international studies. In a U.S.-based survey of 1508 patients, the top concern was rapid relief from itch and pain,^22^ whereas Chinese patients prioritized flare prevention and long-term disease control; symptom relief ranked third. This difference may partly reflect survey design, as our study allowed multiple responses whereas the U.S. survey used single-choice questions. Similarly, a Dutch qualitative study of 15 adolescents with AD found general satisfaction with potent or moderately potent topical corticosteroids, but a desire for faster and more durable effects.^23^

Beyond safety and efficacy, patients also valued attributes such as ease of use, reduced need for long-term treatment or concern about prolonged use, and affordability.

### Efficacy evaluation based on patient preferences

A patient-focused efficacy evaluation should go beyond objective indicators to include symptoms, functional status, and overall well-being. Additional patient preference studies reinforce this need: a 2024 best-worst scaling study of 939 patients with chronic skin diseases (199 with AD), ranked itch, life satisfaction, appearance, body image, and pain as top concerns,^24^ while an earlier study^25^ similarly emphasized psychosocial well-being. These results highlight the need for outcome measures capturing the full impact of disease, not just lesion clearance.

Recent pivotal trials of biologics and JAK inhibitors focused on sustained improvement in skin lesions, itch relief, and quality-of-life, using tools like Eczema Area and Severity Index, Investigator's Global Assessment, and Peak Pruritus Numerical Rating Scale. However, overlapping domains and AD’s complexity limit standardization. Flare frequency, though highly valued by patients, remains underreported and lacks standard measurement. Patient preference data can improve the design of evaluation tools. Flare-up frequency may serve as a key endpoint, alongside reduced medication use, longer dosing intervals, or treatment-free periods.

In parallel, patient-focused endpoints should also reflect treatment sustainability. While new treatments such as IL-4, JAK, or PDE-4 inhibitors are now covered by China’s basic medical insurance system, maintaining disease control often requires prolonged use, and the economic burden from long-term medications remains a prominent concern. Reducing treatment frequency or achieving longer remission periods also aligns with patients’ financial priorities, which highlights the value of patient preference data to efficacy metrics and reimbursement policy.

Long-term follow-up and patient adherence are essential. Lab-based markers, wearable trackers, and remote monitoring systems may improve data quality, but should be applied with attention to privacy and ethics.

### Benefit-risk assessment based on patient preferences

Patient preference data provide insight into acceptable risk levels for meaningful benefit. Our findings show a clear willingness to accept certain risks in exchange for meaningful therapeutic benefits. Even for rare but serious adverse events like malignancy, 30% to 40% patients found the risk acceptable, with tolerance increasing alongside expected benefit. Patients were more accepting of low likelihood or less severity risks, and older or more severe patients showed higher acceptance overall. These insights are particularly relevant to systemic therapies like JAK inhibitors, which offer sustained efficacy^26^ but carry risks like malignancy and serious infections. Many reported are mostly manageable nonmelanoma skin cancers and reversible infections.

Our findings align with international studies. Safety, efficacy, and mode of administration are central to patients’ treatment preferences. A discrete choice experiment across Denmark, France, UK, and Canada found patients with moderate-to-severe AD prioritized avoiding severe adverse events and preferred oral pills over injections, while time to full effect mattered less.^27^ A multi-country qualitative study further emphasized efficacy, speed and duration of symptom relief, side effects, and convenience as major concerns across age groups and severities.^28^ A 2025 systematic review further showed efficacy as the most valued attribute, with patients more willing to accept safety risks for treatment gains.^29^

Together, these studies reinforce our finding that patients are open to accepting some risk, particularly when balanced against meaningful benefits. Patient-informed benefit–risk assessment can help contextualize safety signals and guide measures like labeling, boxed warnings, and targeted monitoring. When benefits outweigh low-frequency risks, such data can support approval decisions and postmarketing strategies to ensure safe use.

### Practical application of patient preference data in drug regulation

This study supports China’s ongoing initiatives to advance patient-focused drug development.^30^, ^31^, ^32^, ^33^ Large-scale surveys and qualitative studies like focus groups are increasingly used, aligning with global trend^34^ toward systematically incorporating patient perspectives across drug lifecycles.

In AD, preference data have already informed technical guidance, especially for JAK inhibitors. Willingness to accept certain risks for faster relief may influence endpoint selection and subgroup analyses. Based on our findings, China’s *Technical Guidelines for Clinical Trials of Atopic Dermatitis Therapeutic Drugs*^35^ identify four core benefit domains: symptoms, signs, long-term disease control, and quality of life. Tools like Eczema Area and Severity Index, Investigator's Global Assessment, Peak Pruritus Numerical Rating Scale, and Dermatology Life Quality Index can capture those changes. Flare-up reduction is also included to track remission duration and relapse.

### Future perspectives

A recent systematic review^36^ highlighted wide heterogeneity in AD preference studies, limiting comparability and regulatory utility. Our study contributes large-scale, real-world data from China, advancing patient-focused benefit–risk assessment and regulatory science. Continued incorporation of patient experience data can help define meaningful endpoints, improve trial relevance, and promote more patient-focused development. A structured framework with clearer standards for data collection, analysis, and regulatory application will enable more responsive development and approval pathways.

### Limitations

This study has several limitations. First, while major dermatology centers were included, the sample was drawn from selected hospitals and patient groups, which may limit generalizability. Second, all data were self-reported and may be subject to recall or reporting bias. Although disease severity was not verified by physicians, the questionnaire adopted clear, predefined criteria referencing common clinical assessment methods, based on lesion extent and impact on quality of life. Moreover, variations in health literacy may have influenced how participants interpreted certain questions. Approximately 70% of respondents had at least a high school education, supporting the reliability and real-world relevance of the data. Third, the cross-sectional design captures preferences at a single time point and may not reflect changes over time, especially as new therapies emerge.

## Conclusions

This nationwide survey reveals how Chinese AD patients weigh benefits and risks. Patients prioritized flare reduction and expressed some tolerance for serious adverse events. Risk tolerance increases with greater expected benefit, while it varies by patient characteristics. These findings support integrating patient preference data into treatment decisions, trial design, and regulatory assessments to align therapeutic strategies with patient needs in China and globally.

### Declaration of generative AI and AI-assisted technologies in the writing process

During the preparation of this work, the authors used ChatGPT (OpenAI, San Francisco, CA) and Grammarly (version 1.116.1.0) in order to assist with language refinement and grammar check. After using this tool, the authors reviewed and edited the content as needed and take full responsibility for the content of the publication.

## Conflicts of interest

None disclosed.
